# An evaluation of the SureID 23comp Human Identification Kit for kinship testing

**DOI:** 10.1038/s41598-019-52838-7

**Published:** 2019-11-14

**Authors:** Hussain M. Alsafiah, Ali A. Aljanabi, Sibte Hadi, Saleh S. Alturayeif, William Goodwin

**Affiliations:** 1 School of Forensic and Applied Sciences, University of Central Lancashire, Preston, United Kingdom; 2 Forensic Genetics Laboratory, General Administration of Criminal Evidences, Public Security, Ministry of Interior, Riyadh, Saudi Arabia

**Keywords:** Genetic markers, DNA recombination

## Abstract

Short tandem repeat (STR) profiling has been routinely used in kinship testing since the introduction of commercial kits in the mid-1990s. While 15 to 23 STR loci normally give definitive results in simple kinship testing, additional loci are sometimes required to resolve complex cases. The SureID 23comp Human Identification Kit, recently released by Health Gene Technologies (China), multiplexes amelogenin and 22 autosomal STRs, 17 of which are non-CODIS STRs. This enables the profiling of 38–40 loci when used in conjunction with widely used commercial kits. In this study, the kit was evaluated for kinship applications as a supplementary STR kit following the minimum criteria for validation recommended by the European Network of Forensic Science Institutes (ENFSI) and the Scientific Working Group on DNA Analysis Methods (SWGDAM) using 500 samples. Performance was comparable with other commercial kits demonstrating: repeatability and reproducibility; precision (maximum s.d. 0.1048 nt); accuracy, all alleles were within ±0.41 nt compared to the actual sizes; heterozygous peak balances at all loci >68%; stutter ratios ranged from 3.8% to 16.15%; full profiles were generated with 125 pg DNA (95.12% of alleles at 62 pg),; and we found 100% concordance over 5 common STRs with the GlobalFiler kit.

## Introduction

The extended number of STR markers required for the Combined DNA Index System (CODIS) and for the European Standard Set (ESS)^1,2^, has led to the development of GlobalFiler PCR Amplification Kit, VeriFiler Plus PCR Amplification Kit (Applied Biosystems (AB), USA), PowerPlex Fusion 6 C System (Promega Corporation, USA) and Investigator 24plex (Qiagen, Germany). The information obtained from these kits will be sufficient in most kinship cases; however, it is still possible to have inconclusive results in complex cases^3^. Kinship testing can be further complicated when the level of consanguinity in the target population is relatively high^4^, or when the family pedigree is deficient^5^.

It has been demonstrated that additional STRs can increase the power of genetic testing in determining the true relationship  between parent-child, siblings or half siblings^6^. For example, Carboni *et al*.^7^ described four complex cases, including incest, which were inconclusive using 13–15 STRs, but that could be resolved using 39–41 STRs.

As most loci are shared between the commonly used kits, the maximum number of STRs that can be tested when combining any two is 24 STRs (e. g. VeriFiler Plus and PowerPlex Fusion 6 C), which necessitates the use of a supplementary STR kit when more loci need to be tested. A set of 25 supplementary STRs was suggested by the National Institute of Standards and Technology (NIST) to increase the certainty in kinship testing^8^; however, no multiplex combing these STRs is commercially available. Supplementary Kits: Microreader 23sp ID^9^ (Suzhou Microread Genetics, China), Goldeneye DNA ID 22NC^10^ (Goldeneye Technology Ltd., China), AGCU 21 + 1^11^ (AGCU ScienTech Incorporation, China) have been developed, but they are only commercially available in China^12^.

Massively parallel systems (MPS) allow simultaneous sequencing of multiple DNA makers. For example, Precision ID GlobalFiler NGS STR^13^ (20 CODIS STRs and nine non-CODIS STRs)(AB), Promega PowerSeq Auto/Y system^14^ (20 CODIS STRs, Penta D, Penta E, and 23 Y-STRs) (Promega Corporation), and ForenSeq DNA Signature Prep^15^ (20 CODIS STRs, seven non-CODIS STRs, 24 Y-STRs, 7X-STRs and 94 identity informative SNPs) (Verogen, USA). These can be utilised to increase the power of kinship testing^15,16^. However, the systems are expensive to establish and are not yet commonly used in many laboratories.

SureID 23 comp Human Identification Kit (Health Gene Technologies, China), combines amelogenin and 22 autosomal STRs: D1S1656, D2S441, D10S1248, D12S391, D16S539 and 17 non-CODIS STRs (D3S1744, D4S2366, D5S2800, D6S474, D7S3048, D8S1132, D9S1122, D11S2368, D13S325, D14S1434, D15S659, D17S1301, D18S1346, D19S253, D20S482, D21S2055, and D22GATA198B05). Twelve of these STRs are not included in other supplementary kits, such as Investigator HDplex Kit^17^ (Qiagen) and PowerPlex CS7 System^18^ (Promega Corporation) (Table 1). The kit is now available in the UK and Europe.

**Table 1:** STR Markers included in the SureID 23comp Kit.

Chr.	SureID 23comp			Investigator HDplex STRs^(a,b)^	PowerPlex CS7 STRs
	STRs^(a)^	Location (GRCh38)	Repeat structure*		
1	D1S1656^a^	230769616–230769683	CCTA [TCTA]n		F13B
2	D2S441^a^	68011947–68011994	[TCTA]n	D2S1360	
3	D3S1744	147374752–147374828	[ATAG]n *atg* [ATAG]n *at* [ATAG]n	D3S1744	
4	D4S2366	6483114–6483172	[GATA]n [GATT]n [GATA]n *gac* [GATA]n	D4S2366	
5	D5S2800	59403132–59403199	[GGTA]n [GACA]n [GATA]n [GATT]n	D5S2500	
6	D6S474	112557951–112558018	[AGAT]n [GATA]n	D6S474, SE33^b^	F13A01
7	D7S3048	21227099–21227174	[TATC]n [TACC]n [CACC]n	D7S1517	
8	D8S1132	106316692–106316774	[TCTA]n *tca* [TCTA]n	D8S1132	LPL
9	D9S1122	77073826–77073873	[TAGA]n		Penta C
10	D10S1248^a^	129294244–129294295	[GGAA]n	D10S2325	
11	D11S2368	19259601–19259684	[TATC]n [TGTC]n [TATC]n		
12	D12S391^a^	12297020–12297095	[AGAT]n [AGAC]n AGAT	D12S391^a^	
13	D13S325	42599304–42599382	[TCTA]n *tca* [TCTA]n		
14	D14S1434	94842054–94842105	[CTGT]n [CTAT]n		
15	D15S659	46081911–46081966	[TATC]n		FESFPS, Penta E
16	D16S539^a^	86352702–86352745	[GATA]n		
17	D17S1301	74684855–74684902	[AGAT]n		
18	D18S1364	65732998–65733056	[TAGA]n TACA [TAGA]n	D18S51^a^	
19	D19S253	15617484–15617531	[ATCT]n		
20	D20S482	4525692–4525747	[AGAT]n		
21	D21S2055	39819508–39819649	[CTAT]n CTAA [CTAT]n *(30n)* [TATC]n	D21S2055	Penta D
22	D22GATA198B05	17169811–17169882	CTCT [ATCT]n [ACCT]n		

This table shows the locations (GRCh38) and repeat structures of the 22 STRs included in the SureID 23comp Kit. Five loci are common with the CODIS and the ESS. Twelve loci are not included in other available supplementary kits (Investigator HDplex and PowerPlex CS7). All information was adopted from^17,18,39^. As proposed by Phillips^19^, the D5S2500 locus name is corrected to D5S2800 (Please see Note S1).

^a^CODIS and ESS locus.

^b^Germany core locus.

*Lowercase and italic nucleotides are not counted in allele nomenclature system^39^.

It is important to note that the D5S2800 locus was named as D5S2500 in the panels and in the supporting documents of the SureID 23comp Kit that may cause some confusion^19^ (Note S1).

Testing additional STRs increases the number of loci situated on the same chromosome (syntenic loci), but raises concerns regarding their independence during meiosis. Syntenic loci are regarded as independent (unlinked) if they are 50 centimorgans (cM) or more apart (at which point the probability of recombination between them is 0.5)^20^. As recombination rates vary along chromosomes, using the physical distance (per bp) may underestimate or overestimate the genetic distance between syntenic loci^21^. Therefore, family studies have been undertaken to estimate the recombination fraction (RF) between syntenic loci^21–24^. However, family studies are expensive and, sometimes, may not be informative enough due to the need of a large number of generations (meiosis) and high percentage of heterozygote genotypes^4,22^. An alternative approach employed by Phillips *et al*.^4^ used the high-density multi-point SNP data of HapMap to approximate the genetic distance between syntenic loci to estimate the RFs, which showed RF values similar to those generated using the family studies.

The SureID 23 comp was used to generate population genetic data for three main populations European, South Asian and African^25^, but it is believed that the kit has not been validated as no publications currently exist, either independently or from the manufacturer. Therefore, this study aimed to evaluate the performance of the SureID 23comp Kit for kinship applications as a supplementary STR kit. The minimum criteria for validation recommended by the ENFSI and by the SWGDAM were followed; mixture studies were not included as the kit is specifically designed to be used in complex kinship testing. In addition, the 17 non-CODIS loci were evaluated using a population (500 individuals) from Saudi Arabia. The data of the 17 non-CODIS loci were merged with the data of 21 STRs generated from the same samples^26^, and were used for testing potential linkage between syntenic loci (linkage disequilibrium, LD). RF values of syntenic pairs were reviewed from^4,12,21,22,24^, which were estimated using family studies and using HapMap data. RFs derived from HapMap data for D2S1338-D2S441, TPOX-D2S1338, FGA-D4S2366 and D5S2800-CSF1PO pairs, were calculated based on cumulative genetic map distance provided by Phillips^12^ and using the Excel tool developed by Phillips *et al*.^4^.

## Methods

The study was performed in accordance with the ethical guidelines of the University of Central Lancashire (UK) and was approved by the Security Forces Hospitals Programme (SFHP, Saudi Arabia), the Medical Legal Directorate, Ministry of Health (MLD, Iraq) and by Ethics Committee of the University of Central Lancashire.

### DNA samples

Initial tests of the SureID 23comp Kit were carried out using the 2800 M control DNA (Promega Corporation). The control DNA was also used for sensitivity and stochastic tests by preparing five serial dilutions of (500, 250, 125, 62, and 31) pg. In addition, 0.5 ng of the control were amplified with the presence of different concentrations (50, 75, 100, 120 and 150) ng/µl of common PCR inhibitors humic and tannic acids (Sigma-Aldrich, USA). The 9947 A control DNA provided with the kit was used with every run as a positive control.

The performance of the kit was further assessed using nine bone samples that were collected from a mass grave in Iraq after obtaining a permission from the MLD, Ministry of Health (Iraq). The bone samples were previously extracted using PrepFiler BTA Forensic DNA Extraction Kit (AB) and were quantified using Quantifiler Trio DNA Quantification Kit (AB). The concentrations of the small fragments of the Quantifiler Trio ranged from 0.0173 ng/µl to 0.3271 ng/µl and the Degradation Indexes (DI) were from 1.6758 to 57.666 (Table S1). These samples were previously profiled using one or more of the commonly used STR kits (Table S1).

The study of precision, accuracy, peak balance, concordance and stutter peak ratios were carried out using 500 samples from unrelated individuals from the population of Saudi Arabia. The samples were collected after obtaining informed consents from all participants. The extraction and the quantification were described in a previous study that evaluated the 21 STR loci included in the GlobalFiler PCR Amplification Kit^26^.

### Sample amplification

The Initial tests of the SureID 23comp used two reaction volumes that were optimised by the manufacturer. A 25 µl volume that contained 12.5 µl master mix, 6.25 µl primer mix and up to 6.25 µl of DNA template; and a 10 µl volume that contained 5 µl master mix, 2.5 µl primer mix and up to 2.5 µl of DNA template. The range of recommended template DNA is 0.5–4 ng. To validate both volumes, two operators carried out the initial tests independently with 0.5 ng of control DNA in five replicates (20 tests in total).

Three DNA concentrations (0.5, 0.35, and 0.25) ng were used for the first 90 samples from the Saudi population to evaluate the performance of the 10 µl volume. Then, the rest of samples (410 samples) were genotyped using the 10 µl volume with 0.5 ng DNA input per reaction.

Microamp Optical 96-Well Reaction Plates and Microamp Optical Adhesive Films (AB), were used for amplification; 2.5 µl of the DNA and DNase/RNase-free water were added to 7.5 µl of the SureID 23comp mix (5 µl master mix and 2.5 µl primer mix).

A Veriti thermal cycler (AB) was employed to carry out the amplification reactions as follows: [95 °C (5 min)]/[94 °C (10 s) 61 °C (60 s) 70 °C (30 s)] 28–30 cycles/[60 °C (15 min)]. The 28 cycle protocol was used for the initial tests, stability tests and for the 500 samples. For sensitivity and stochastic study, the serial dilution samples were amplified in five replicates using both reaction volumes, each volume was tested with 28 and 30 PCR cycles (100 tests in total). For the bone samples, the 25 µl volume and 30 PCR cycles were used.

### DNA separation and analysis

An ABI 3500 DNA Genetic Analyser with 50 cm capillaries and POP-6 polymer (AB) was used for the separation and detection. The spectral calibration mix was prepared by adding 8 µl HGT 5-Dye Matrix Standard (Health Gene Technologies) to  200 µl of Hi-Di Formamide (AB); 10 µl were dispensed to each well. In the data collection software (AB), the dye set of SureID 23comp was created based on the G5 template as recommended by manufacturer.

Samples were prepared for separation and detection by adding 1 µl of PCR products or of an allelic ladder (Health Gene Technologies) to 9 µl of Hi-Di Formamide/Size-500-Plus Mix. This mix was prepared by 9 µl of Hi-Di Formamide (AB) and 0.25 µl Size-500-Plus (Health Gene Technologies), for each sample. Based on the manufacturer’s guidelines, the run time in the module HID36_POP4 should be 1,210–1,500 s when using a 36 cm capillary. In this study, the run time was increased to 3900 s to accommodate the use of the 50 cm capillaries.

Alleles from the 23 markers were called using GeneMapper *ID-X* Software v1.2 (AB) with an allelic ladder mix supported by panels and bins provided by the manufacturer. For the sensitivity and stability tests, the minimum relative fluorescent units (RFU) was 50 RFU for heterozygous genotypes and was 150 RFU for homozygous genotypes.

### Population study

PowerStat v 1.2 (Promega Corporation) was utilised to calculate allele frequencies, match probability, power of discrimination, power of exclusion, typical paternity index and polymorphic information contents. RStudio platform v1.2.1335^27^ and DNA tools package v0.1-22^28^, were used to identify the maximum number of matched loci within the 500 samples. The Exact test for detecting deviation from the Hardy-Weinberg equilibrium (HWE) was carried out by Arlequin v3.5.2.1 Software^29^, using 1,000,000 steps for the Markov chain and 100,000 for the dememorization steps.

### LD and RF study

The Arlequin v3.5.2.1 Software^29^ was also used to test LD between potentially 18 syntenic loci resulting from combining 38 loci available in GlobalFiler (21 STRs) and SureID 23comp (17 non-CODIS STRs) kits. This was carried out by applying 1000 in the permutations and 2 in the Expectation-Maximisation (EM) algorithm. RF values of syntenic pairs were reviewed from^4,12,21,22,24^, which were estimated using family studies and using HapMap data. RFs derived from HapMap data for D2S1338-D2S441, TPOX-D2S1338, FGA-D4S2366 and D5S2800-CSF1PO pairs, were calculated based on cumulative genetic map distance provided by Phillips[12] and using the Excel tool developed by Phillips *et al*.^4^.

## Results and Discussion

### Evaluation of the SureID 23comp Kit

The first step in the validation was a confirmation of the identity of the D5 locus included in the multiplex by testing the 9947 A control DNA provided in the kit as a positive control (Note 1). The 9947 A control DNA showed alleles 14, 23 at the D5 locus confirming that the locus is D5S2800 and not D5S2500^19^.

In the initial tests of the SureID 23comp, the replicates of the 2800 M control DNA, were successfully profiled by two independent operators and 20 replicates were fully concordant confirming the repeatability and reproducibility.

**Figure 1:**
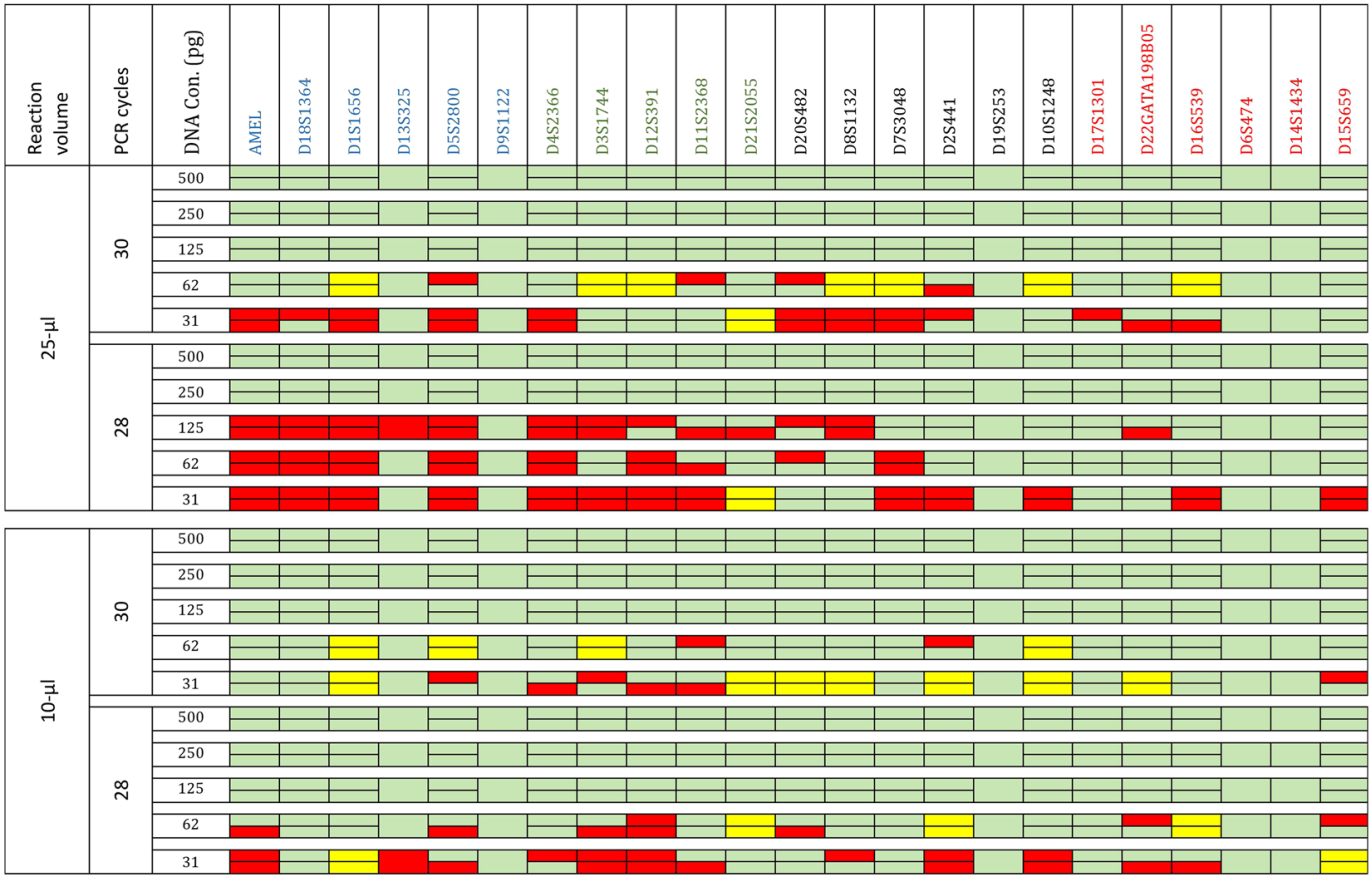
Sensitivity and stochastic tests for the SureID 23comp Kit. Serial dilutions (500, 250, 125, 62, and 31) pg were prepared from the 2800 M control DNA (Promega Corporation). Each test was done on five replicates and the sample with the highest number of detected alleles are shown. Each cell represents an allele and merged cells represent homozygote loci in the 2800 M. Green cells identify detected alleles with ≥60% peak balance ratios. Yellow cells identify detected alleles with <60% peak balance ratios. Red cells represent undetected alleles with threshold of 50 RFU/150 RFU for heterozygotes/homozygotes.

Each of the dilution series samples was replicated five times using the 25 µl and 10 µl volumes with 28 and 30 cycles (100 reactions in total). Full profiles were generated from the 125 pg samples when using the 10 µl volume (28 and 30 cycles), while 25 µl volume was able to generate a full profile with 30 PCR cycles only. For the 62 pg samples, the 28 cycle protocol allowed detection of 60.9% (25 µl) to 80.48% (10 µl) and the 30 cycle protocol allowed detection of 90.2% (25 µl) to 95.12% (10 µl) where the rest of the alleles were visible and could be detected with a reduced RFU threshold of 30. With 31pg, the profile percentage was from 53.6% (25 µl) to 85.3% (10 µl) using the 30 cycle protocol, while allele dropout was observed with 28 cycle protocol (Fig. 1). The sensitivity results are comparable to other commonly used kits, for example, Identifiler Kit^30^, Investigator HDplex Kit^21^ and PowerPlex Fusion 6 C System^31^ where the profile percentage ranged from 82% to 94% for the 62 pg and from 37% to 72% for the 31pg.

The performance of the SureID 23comp Kit with different concentrations of two common PCR inhibitors was tested. Full profiles were generated in the presence of ≤ 120 ng/µl of tannic acid and of ≤ 75 ng/µl of humic acid (Figs S1 and S2). Although these levels are similar to those reported for the SureID PanGlobal Kit (Health Gene Technologies)^32^, other commonly used kits are more robust in the presence of higher concentrations of inhibitors^33^ (Fig. S3).

The sensitivity and the stability of the SureID 23comp were further evaluated using nine bone samples. The bone samples were profiled using the 25 µl volume to increase the volume of the DNA input in the PCRs to 6.25 µl. Seven samples, where the total DNA input ranged from 0.2575 ng to 2.0444 ng/reaction, showed similar percentage of detected alleles to other kits previously used (Table S1). However, two samples that had lower concentrations and higher DIs of 0.0173 ng/µl (DI: 57.7) and 0.0194 ng/µl (DI: 16.2) (total DNA input 0.1081 ng and 0.1213 ng), the performance deteriorated, both in absolute terms, and in comparison to other kits. It is notable that the capacity of DNA quantity in the other kits (15 µl) allowed 2.4 fold more DNA to be added to the reaction compared to the SureID 23comp (6.25 µl). Overall, the sensitivity tests when using the DNA control, demonstrated the robustness of generating full profiles even below the recommended DNA concentrations and showed similar sensitivity to other commonly used STR kits. However, this kit  demonstrated reduced sensitivity with the bone samples, which is most likely due to the limited capacity of DNA input compared to other kits (Table S2). Although this kit was designed as a supplementary kit for forensic genetics laboratories, some cases may involve human remains (e.g. disaster victim identification (DVI)). Therefore, increasing the concentrations of the master and primer mixes (e.g. to 2X) would permit additional space for more DNA input especially for highly degraded samples.

**Figure 2:**
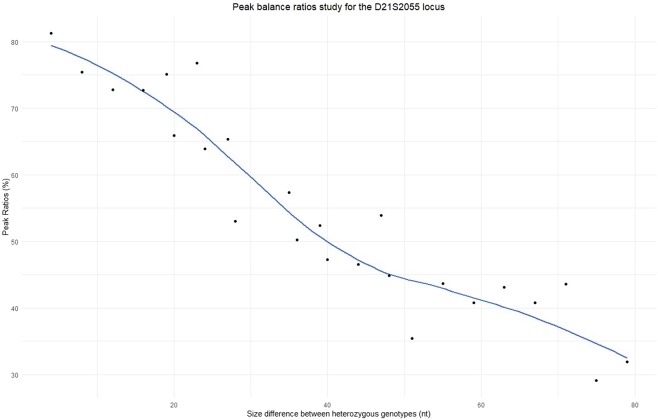
Peak balance ratios study for the D21S2055 locus. This figure shows a study of the correlation between the size differences between heterozygous alleles and the peak balance ratio for the D21S2055 locus using data of 500 samples. The peak ratios of all genotypes that have the same size difference (nt) (e.g. the genotypes 13, 17; 14, 18; and 15, 19 have the same size difference of 4 nt) were averaged and are represented by the black dots. The blue line shows the smoothed mean of the peak ratios. Heterozygote alleles with >50 nucleotides differences showed peak ratios <45%.

Peak balances evaluation started with measuring of the optimal DNA quantity for the 10 µl volume using the first 90 samples that were tested using three different DNA quantities (0.5, 0.35, and 0.25) ng. With all template amounts the minimum peak balance ratios were >68%, which meets the criteria set out in the ENFSI guidelines (>60%). The DNA input of 0.5 ng achieved the most balanced heterozygous peaks with an average of 88.31% (Table S3), the values were which are similar to ratios observed when testing other kits, for example Investigator HDplex Kit^21^. The D21S2055 showed the lowest degree of balance at all template concentrations with ratios of 73.11% at 0.5 ng, 79.75% at 0.35 ng and 68.12% at 0.25 ng (Table S3). The remaining 410 samples were successfully profiled using the 10 µl volume and 0.5 ng of DNA input. Overall, the intra-locus balances were 81.8% (D21S2055) - 96.9% (D16S539), the intra-dye balances 71.9% (TAMRA) – 82.6% (JOE), and the inter-dye balances >43%. These figures are consistent with the recommended levels that are >70% for intra-locus balance, 50% for intra-dye balance, and >30% for inter-dye balance^32^. The peak imbalances of the D21S2055 became less than 50% when the size difference between heterozygous alleles was more than ten repeats (40 nt) (Fig. 2). The peak balances further decreased to <45% when the size difference became >50 nt (>12 repeats). For example, an average of 43.5% for the genotypes (16.1, 34) (two samples), and 31.8% for the genotype (16.1, 36) (one sample) (Fig. 2). This locus is the longest marker in this kit (332 bp to 420 bp) and has the highest number of possible alleles (23 alleles: 16.1 to 38).

**Figure 3:**
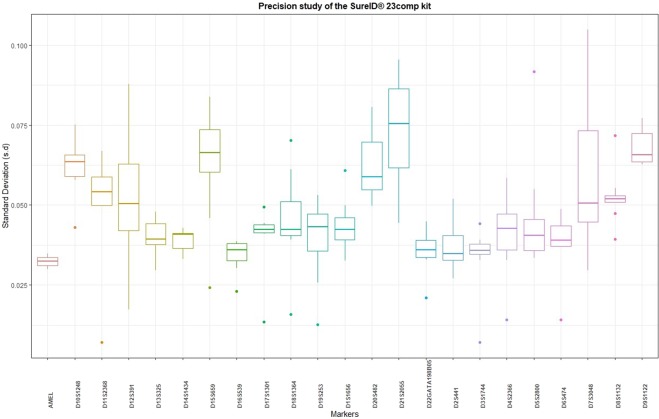
Precision study of the SureID 23comp Kit. The figure shows standard deviation (s.d) values of the fragment sizes of 22,981 alleles generated from 500 samples tested by the SureID 23comp. The highest s.d. was observed in allele 21 at D7S3048 (0.1048 nt).

**Figure 4:**
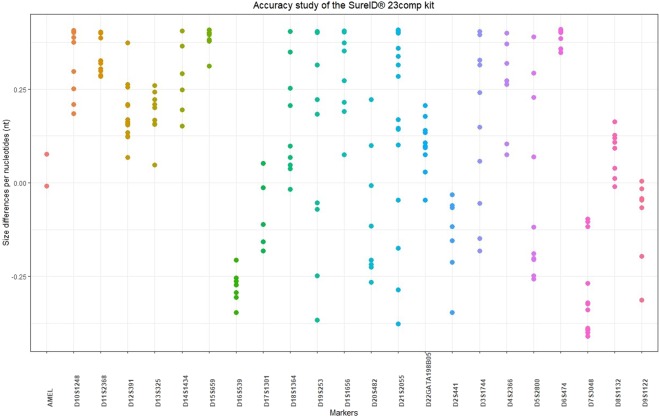
Accuracy study of the SureID 23comp Kit. The average of the size values of each allele in the data of the 500 samples and in 21 allelic ladders were compared to the actual sizes of the corresponding allele (actual sizes provided by the manufacturer). The size differences per nucleotides were calculated and are represented by the coloured dots. All alleles fell within the range of ±0.41 nt of the allelic widow; the largest differences were seen at D6S474 allele 17 (0.4096 nt) and D7S3048 allele 26 (−0.4084 nt).

For the precision study, the data of 22,975 alleles (23,000 alleles from 500 samples excluding 25 alleles with a single observation) were used to calculate the standard deviation (s.d.) of the fragment sizes of each allele at a locus. Overall, the maximum s.d. was 0.1048 nucleotide (nt) observed in allele 21 at D7S3048 and the minimum was 0.0071 nt observed in allele 22 at D3S1744 (Fig. 3). To measure the accuracy of the kit, the average sizes of each allele in the data of the 500 samples and in 21 allelic ladders  were compared to the actual size values of the corresponding allele (actual sizes provided by the manufacturer). All alleles fell within the range of ±0.41 nt, where allele 17 at D6S474 (0.4096 nt) and allele 26 at D7S3048 (−0.4084 nt) recorded the highest difference comparing to the actual sizes (Fig. 4). The precision and the accuracy tests demonstrated the capability of detecting heterozygous alleles that differ by a single nucleotide and demonstrate that it is unlikely for any allele to be sized out of the designated window (±0.5 nt). The SureID 23comp was reliably able to detect genotypes where the difference between the alleles was a single nucleotide, for example (11.3, 12) at D2S441, (15.3, 16) and (16.3, 17) at D1S1656 (100% concordant with GlobalFiler genotypes).

Stutter artefacts are common to all PCR-based STR analysis and the most common type of stutter is a peak with one repeat smaller than the true allele^34^. In this study, the average of the stutter peak ratios was 9.18% and the range was from 3.8% for D2S441 to 16.15% for D12S391 (Fig. S4).

**Figure 5:**
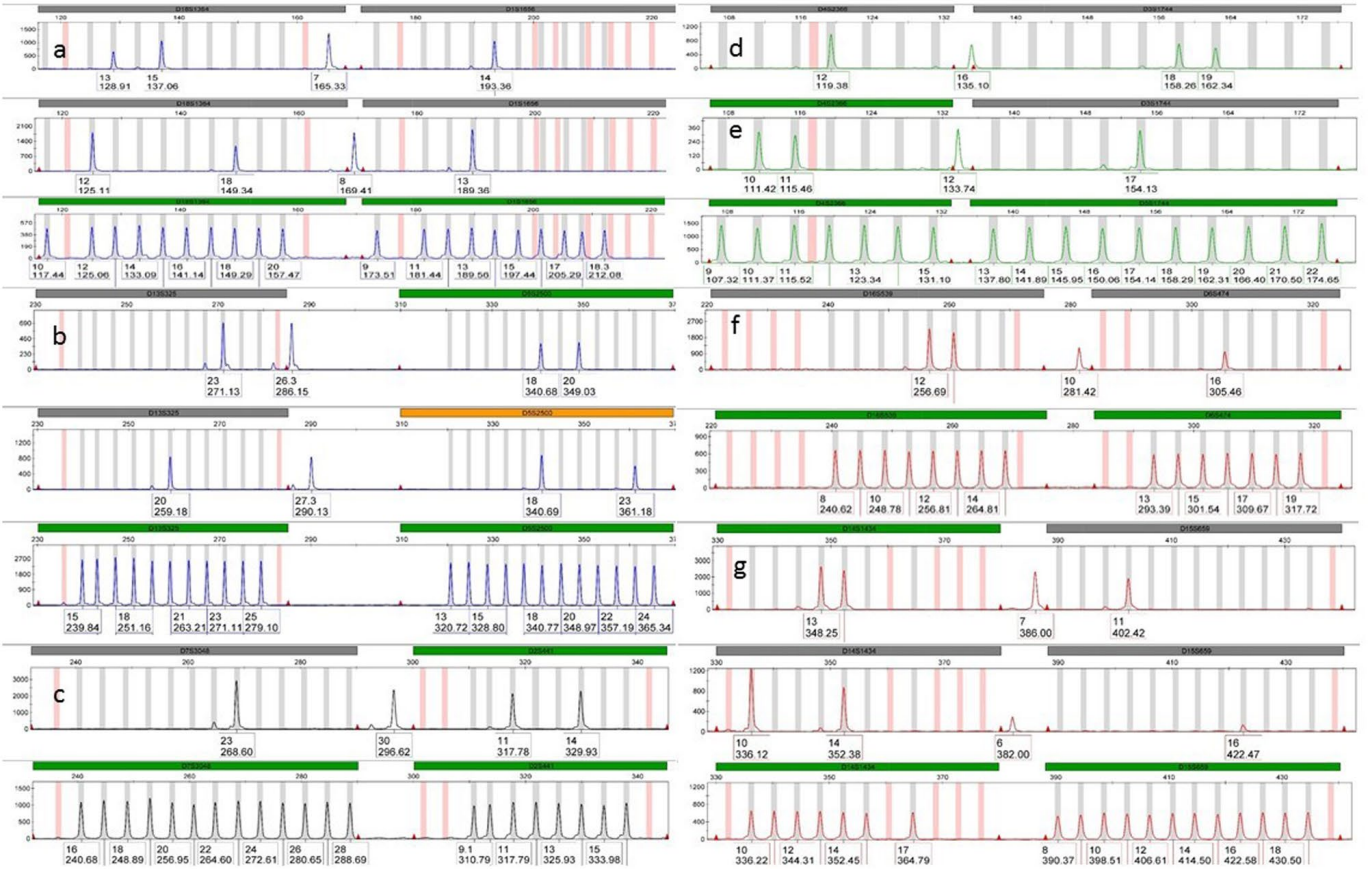
Alleles outside the allelic ladder windows of the SureID 23comp Kit. This figure shows ten alleles observed in the population of Saudi Arabia that are not represented in the allelic ladder and were situated outside the designated widow of their loci: (**a**) Alleles 7 and 8 at D1S1656; (**b**) Alleles 26.3 and 27.3 at D13S325; (**c**) Allele 30 at D7S3048; (**d**) Allele 16 at D4S2366; (**e**) Allele 12 at D3S1744; (**f**) Allele 10 at D6S474; (**g**) Alleles 6 and 7 at D15S659. Allele 7 at D1S1656 (**a**) was situated under the designated area of D18S1364 locus.

This kit provides an allelic ladder representing 232 alleles that are supported by 53 additional bins for variant alleles (Fig. S5). After analysing the 500 samples, 34 alleles in 15 loci were not represented by the allelic ladder, three of which had been observed ≥40 times (Table 2). In addition, ten of these alleles were situated outside the designated widow of their loci: alleles 7 and 8 at D1S1656, 26.3 and 27.3 at D13S325, allele 16 at D4S2366, allele 12 at D3S1744, allele 30 at D7S3048, allele 10 at D6S474 and alleles 6 and 7 at D15S659 (Fig. 5a–g). One allele (7 at D1S1656) was situated under the designated area of D18S1364 locus (Fig. 5a). Although this allele could, in principle, belong to D18S1364 forming triplet allele genotype, it was confirmed by sequencing that it belongs to D1S1656^35^. It is not necessary for an allelic ladder to represent all rare alleles, however, alleles outside the designated window of a locus may be misinterpreted especially when adjacent loci are homozygous. Examining data of 256 samples collected from the population of Ningbo, China (data provided by the Health Gene Technologies) (Table 2), most alleles present in the Saudi Arabian population but not present in the allelic ladder were absent in the Ningbo population.

**Table 2:** Alleles not represented by the allelic ladder of SureID 23comp Kit detected in the population of Saudi Arabia.

STRs	Allele	frequency		STRs	Allele	frequency	
		Saudi	Ningbo			Saudi	Ningbo
D18S1364	11	0.001	0.002	D13S325	26.3	0.002	0
D1S1656	7	0.001	0		27.3	0.001	0
	8	0.001	0	D8S1132	13.1	0.001	0
	10	0.004	0.001		15	0.003	0
	14.3	0.002	0	D7S3048	30	0.001	0
	15.3	0.040	0	D2S441	8.3	0.001	0
	16.3	0.061	0.007		9	0.005	0
	18	0.003	0.011		11.3	0.066	0
	19.3	0.006	0.003		13.3	0.001	0
	20.3	0.001	0.002	D19S253	6	0.004	0
D9S1122	7	0.001	0		16	0.001	0
D4S2366	16	0.002	0	D22GATA198B05	11.2	0.001	0
D3S1744	12	0.001	0		12	0.004	0
D12S391	18.3	0.005	0	D6S474	10	0.001	0
	19.1	0.001	0	D14S1434	16	0.004	0.004
	19.3	0.004	0	D15S659	6	0.001	0
	27	0.003	0.003		7	0.003	0

Thirty-four alleles were detected at 15 STRs that are not represented by the allelic ladder of the kit. It shows also the frequency of these alleles in Ningbo population (data provided by the Health Gene Technologies) and in the population of Saudi Arabia. The frequencies of detected alleles in the population of Saudi Arabia ranged from 0.001 (one observation) to 0.066 (66 observations).

The concordance study was also carried out by comparing data of the 500 samples obtained from this study and that generated using the GlobalFiler kit^26^. The five common loci (D1S1656, D2S441, D10S1248, D12S391 and D16S539) showed 100% concordance. In addition, alleles generated from the bone samples using the SureID 23comp Kit at the common loci were concordant with alleles generated using the other kits. In addition, the amelogenin showed concordant genotypes to those generated by the GlobalFiler kit.

### Population study

Assuming independence, the combined match probability (CMP) for the 22 STRs was 7.4 × 10^−27^, and the combined power of exclusion (CPE) was 0.999998692. D21S2055 was the most informative locus with a match probability (MP) of 0.016, and D17S1301 was the least informative locus with a MP of 0.162. Heterozygosity ranged from 0.624 (D20S482) to 0.89 (D21S2055). The number of observed alleles per locus varied from 7 alleles in D17S1301 to 20 alleles in D21S2055. Three alleles, allele 14 in D20S482, allele 12 in D17S1301 and allele 12 in D9S1122; showed very high frequencies of 0.477, 0.450 and 0.405 respectively. The frequency of the theoretical most common SureID DNA profile, generated based on the frequencies of the 22 STRs (and assuming heterozygosity) was 3 × 10^−21^ that equates to 1 in 3.3 × 10^20^ (Table S4). The CMP of the 22 STRs (7.4 × 10^−27^) is greater than CMP calculated when using the 21 loci of GlobalFiler kit (1.421 × 10^−26^)^26^. Apart of SE33, the SureID 23comp Kit includes the four most informative loci that have been studied for the population of Saudi Arabia (D21S2055, D12S391, D7S3048, and D1S1656), two of which are included in the GlobalFiler kit (Table S4). Data of the 17 non-CODIS loci was merged to the data of 21 loci (total 38 loci) and was statistically evaluated for the population of Saudi Arabia. Assuming independence, the 38 loci combined provided 1.7 × 10^−46^ CMP, and 0.999999999934248 CPE, and the theoretically most common DNA profile (assuming heterozygosity at all loci) was 2.7 × 10^−36^, which equates to 1 in 3.7 × 1035 (Table S4).

The 22 loci were in HWE (*P*-value > 0.05) except D20S482 which showed significant deviation (*P-*value = 0). For the Saudi population, therefore, this locus should not be included in the product rule to calculate the probability of a DNA profile.

The data of the 22 loci of the SureID 23comp was uploaded to the R studio to find out the maximum number of matched loci between any two DNA profiles using the DNA Tools package. In the 500 samples, the maximum number of loci matching between any two samples was 9 out of 22 loci (40% of the 22 loci), which was observed in two sample pairs. One pair of profiles showed partial matching (i.e. one of the two alleles) at 20 out of 22 loci. This illustrates the power of the additional loci for human identification and kinship testing (Table S5).

To assess the SureID 23comp for kinship testing based on the typical paternity index, the combined typical paternity index (CPI) was used to calculate the paternity probabilities with different prior probabilities (Pr): 0.90, 0.50 and 0.10. Assuming that all loci are independent, the probabilities of paternity were 99.99999988% (Pr = 0.90), 99.99999893% (Pr = 0.50) and 99.99999041% (Pr = 0.10), which are higher than those probabilities calculated when using the GlobalFiler kit (Table S6). Under the same assumptions, the 38 loci increased the probabilities of paternity to 99,9999999999999972% (Pr = 0.90), 99,9999999999999750% (Pr = 0.50) and to 99,9999999999997750% (Pr = 0.10) (Table S6).

The ForenSeq DNA Signature Prep (Verogen), when combining the 94 SNPs and the 27 STRs, has shown much higher CMP of 10^−67^–10^−69^ (length-based STRs calls) and of (10^71^–10^−74^) (sequence-based STRs calls), where the CMP of the 94 SNPs alone were (10^−38^–10^−35^)^36^. In addition, using MPS systems in kinship testing could help in tracking mismatches between tested individuals that have occurred due to mutation in the binding sites of primers^15^. However, this requires additional technology to be implement and is currently not available in many countries.

Five loci included in the SureID 23comp (D18S1364, D4S2366, D3S1744, D20S482, and D17S1301) were designed to have amplicons <200 bp facilitating characterisation of partial degraded samples. In addition, the primer pairs of the D2S441 and D10S1248 mini-STRs, in the SureID 23comp Kit, were designed to generate longer amplicons (310–446 bp) than primer pairs used in the GlobalFiler kit (<125 bp), which enabled more space for other mini-STRs, e. g. D17S1301 and D4S2366 (<150 bp) in the SureID 23comp. As a result, combining both kits will increase the number of STRs <200 bp to 12 STRs and thus increases the CMP to 7.9 × 10^−14^ (Table S7).

### LD and RF study

Family studies have previously been carried out to estimate recombination fraction (RF) for four syntenic loci residing on the same chromosome’s arm vWA-D12S391, D5S818-CSF1PO, TPOX-D2S441 and D21S11-PentaD typed when using any of the commonly used STR kits^21–23^. RF values were 0.17^21^, 0.13^22^, and 0.11^23^ for vWA-D12S391; 0.197^37^ for D5S818-CSF1PO; 0.53^21^ for TPOX-D2S441 and 0.316^37^ D21S11-PentaD. The high-density multi-point SNP data of HapMap was also used to approximate the genetic distance between these syntenic loci and gave RF values of 0.12 for vWA-D12S391, 0.25 for D5S818-CSF1PO, 0.36 for D21S11-PentaD, and of 0.47 for TPOX-D2S441^4^; these values are similar to those generated using family studies.

It was concluded that, for most pedigrees, the RF value of ~0.12 has almost zero effect in any population as long as no linkage disequilibrium was detected^38^. For some pedigrees, where at least one individual has a heterozygote genotype in the both syntenic loci and is involved in at least two transmissions of genetic components, linkage can have a significant effect in the product rule calculation in kinship testing^38^.

However, using the SureID 23comp Kit in conjunction with any of commonly used kits, will increase the number of potentially linked syntenic loci located on the same arm to 12–15 pairs (D5S818-D5S2800, CSF1PO-D5S2800, SE33-D6S474, D8S1179-D8S1132, TH01-D11S2368, D13S317-D13S325, Penta E-D15S659, D18S51-D18S1364, D21S2055-D21S11, D21S2055-Penta D, D22S1045- D22GATA198B05, vWA-D12S391, D5S818-CSF1PO, TPOX-D2S441 and D21S11-PentaD).

The potential linkage between 18 syntenic loci (12 on the same arm and 6 on different arms), which resulted from combining the 38 STRs, were tested using the likelihood ratio test (because the gamatic phase of the genotypes is unknown)^29^. Although, significant departure from HWE could invalidate the test, none of the 18 pairs is on chromosome 20 and thus the significant departure of D20S482 from HWE is not relevant. After Bonferroni correction (*P*-value = 0.003), no significant deviation from linkage equilibrium (LE) was detected between any of the 18 syntenic loci (Table 3).

**Table 3:** LD test results for 18 pairs of syntenic loci formed when combining loci included in the SureID 23comp and GlobalFiler kits.

Chr.	Syntenic Pair	LD test *P*-value	HapMap proxy SNP position (GRCh37)^b^	Cumulative genetic map distance in cM^b^	Genetic map distance in cM	RFs from Kosambi mapping function^a–c^	RFs estimated based on family studies^d–f^
Chr.2	TPOX	0.97764	1493487	1.6661	88.8129	0.4721^a^	0.53^d^
	D2S441		68239020	90.47903			
Chr.2(p-q)	D2S1338	0.99164	218879435	223.4832	133.0042	0.4951^c^	0.58^d^
	D2S441		68239020	90.47903			
Chr.2(p-q)	TPOX	0.79338	1493487	1.6661	221.8171	0.4999^c^	0.51^d^
	D2S1338		218879435	223.4832			
Chr.3(p-q)	D3S1358	0.99193	45582627	67.1789	90.0624	0.4735^a^	0.64^d^
	D3S1744		147092143	157.24131			
Chr.4(p-q)	FGA	0.99771	155508100	156.81293	143.8662	0.4968^c^	0.51^d^
	D4S2366		6484806	12.9467			
Chr.5	D5S2800	0.15198	58698677	70.3208	56.3520	0.4050^b^	N/A
	D5S818		123111652	126.67284			
Chr.5	D5S2800	0.85646	58698677	70.3208	84.1132	0.4666^c^	N/A
	CSF1PO		149455757	154.43395			
Chr.5	D5S818	0.69008	123111652	126.67284	27.7611	0.2522^a^	0.18^d^
	CSF1PO		149455757	154.43395			
Chr.6	SE33	0.99963	88986609	95.44921	23.2133	0.2168^a^	0.19^d^
	D6S474		112879893	118.66248			
Chr.7(p-q)	D7S3048	0.98684	21266723	36.14071	64.0605	0.4284^b^	0.4997^e^
	D7S820		83789257	100.2012			
Chr.8	D8S1132	0.23577	107330479	119.96228	16.4809	0.1591^a^	0.1443^e^
	D8S1179		125907927	136.44313			
Chr.11	TH01	0.20421	2192166	4.48933	28.3996	0.2569^b^	0.2152^e^
	D11S2368		19281171	32.88891			
Chr.12	vWA	0.89307	6093924	15.63031	11.9410	0.1172^a^	0.1259^e^
	D12S391		12450501	27.57129			
Chr.13	D13S325	0.97422	43173444	44.90825	34.9225	0.3016^b^	0.2533^e^
	D13S317		82721723	79.83074			
Chr.18	D18S51	0.04312	60949983	88.92051	2.2970	0.0229^b^	0.0327^e^
	D18S1364		63400151	91.21746			
Chr.19(p-q)	D19S433	0.78742	30417603	51.72618	12.4538	0.1228^b^	0.1101^f^
	D19S253		15728103	39.27234			
Chr.21	D21S11	1	20554558	14.64555	34.8192	0.3010^a^	0.32^d^
	D21S2055		41191871	49.46478			
Chr.22	D22S1045	0.98893	37535663	46.21362	38.8178	0.3253^b^	N/A
	D22GATA198B05		17651831	7.39585			
Chr.15*	Penta E	—	97377441	124.05054	74.5331	0.4517^b^	N/A
	D15S659		46371620	49.51748			
Chr.21*	Penta D	—	45056178	59.37591	9.9111	0.0978^b^	N/A
	D21S2055		41191871	49.46478			

This test was performed for data generated from the 500 samples from Saudi population for the 38 loci. No significant deviation from linkage equilibrium (LE) was detected (*P*-value = 0.003). RFs that previously derived from HapMap data and from family studies were reviewed from literature^4,12,21,22,24^. RFs derived from HapMap data for D2S1338-D2S441, TPOX-D2S1338, FGA-D4S2366 and D5S2800-CSF1PO pairs, were calculated based on cumulative genetic map distance provided in Phillips^12^ and using Excel tool developed by Phillips *et al*.^4^.

^a^Data reviewed from Phillips *et al*.^4^^.^

^b^Data reviewed from Phillips^12^^.^

^c^RFs were calculated based on cumulative genetic map distance provided in Phillips^12^ and using an excel tool developed by Phillips *et al*.^4^.

^d^Data reviewed from Westen *et al*.^21^.

^e^Data reviewed from Liu *et al*.^22^.

^f^Data reviewed from Wu *et al*.^24^.

*Potential syntenic pairs when combining the SureID 23comp with kits that include the Penta E and Penta D loci (LD was not tested).

N/A: No data available from family studies.

Table 3 also reviews RFs that previously derived from HapMap data and from family studies^4,12,21,22,24^. In addition, RFs derived from HapMap data for D2S1338-D2S441, TPOX-D2S1338, FGA-D4S2366 and D5S2800-CSF1PO pairs, were calculated based on cumulative genetic map distance provided in Phillips^12^ and using the Excel tool developed by Phillips *et al*.^4^.

The genetic distance (cM) estimated based on the HapMap data ranged from 3.49 cM for D18S51-D18S1364 pair to 226.5 cM for TPOX-D2S1338 pair. Apart from the D18S51-D18S1364 and PentaD-D21S2055 pairs, all potential linked loci showed RF values > 0.12 and thus they can be included in the product rule calculation for kinship testing for most pedigrees. For pairs showing RF values <0.12, excluding the less informative locus from the probability estimation^23^, which is the D18S1364 for the Saudi population, is an option. However, due to concerns that this may lead to an overestimation or to an underestimation of the strength of an evidence, Gill *et al*.^38^ illustrated a methodology by which the RFs can be included in the probability estimation.

## Conclusion

The SureID 23comp was validated following the minimum criteria for validation recommended by the ENFSI and the SWGDAM for forensic applications as a supplementary STR kit. The kit is reproducible, precise, accurate and reliable for forensic application as a supplementary kit and for databasing. The identity of the D5 locus was confirmed and has now been updated by the manufacturer to D5S2800.

The sensitivity tests demonstrate the capability of generating a full profile below the recommended DNA input but showed that the kit was less sensitive compared to other commonly with degraded samples, which was at least in part because of the lower volume of template that can be added. Therefore, increasing the concentration of the reaction mix will allow more space for DNA input to 15 µl rather than 6.25 µl, which will increase the sensitivity of the kit.

Including additional alleles and allele variations in the available spaces of the allelic ladder will allow specific allele designation and will minimise the need to re-run undesignated alleles. In addition, caution should be taken when using the kit with potentially degraded samples (e.g. DVI cases) due to the peak imbalance of D21S2055.

For kinship testing, typically, the kit achieved a CPI of 93835307.21 that is two times higher the CPI recorded for the GlobalFiler kit and allowed higher paternity probabilities 99.99999988%, 99.99999893% and 99.99999041% in different prior probabilities, demonstrating the informativeness of the STRs included in the kit. In addition, utilising the 38 loci will increase the certainty of the paternity probabilities, using different prior probabilities, to 99,9999999999999972%, 99,9999999999999750% and to 99,9999999999997750%, which is useful for complex cases. However, caution should be considered when estimating likelihoods if the D18S51-D18S1364 and/or PentaD-D21S2055 pairs are included.

Overall, this study evaluates the utility of the SureID 23comp as a supplementary kit for kinship testing and determined that the kit met the criteria commonly used in forensic genetics laboratories. The kit allows the analysis of 17 non-CODIS loci and increases likelihood ratios, and thereby has the potential to increase the level of confidence in conclusions in complex kinship tests using already established technology.

## Supplementary information

Supplementary Information

## Data Availability

Data can be requested via: whgoodwin@uclan.ac.uk or hmhalsafiah@uclan.ac.uk.
