# Supplementary material for: An evaluation of the SureID 23comp Human Identification Kit for kinship testing
Source: Sci Rep. 2019 Nov 14;9:16859. doi: 10.1038/s41598-019-52838-7 (PMC6856129; doi:10.1038/s41598-019-52838-7)
Supplement: Supplementary file 1 — Supplementary Information [file 41598_2019_52838_MOESM1_ESM.pdf]

An evaluation of the SureID 23comp Human Identification Kit for kinship testing.

Hussain M. Alsafiah [1, 2] *, Ali Aljanabi [1], Sibte Hadi. [1], Saleh S. Alturayeif [2], Will Goodwin [1] *

[1] *School of Forensic and Applied Sciences, University of Central Lancashire, Preston, United Kingdom.*

[2] *Forensic Genetics Laboratory, General Administration of Criminal Evidences, Public Security, Ministry of Interior, Kingdom of Saudi Arabia.*

Note S 1. D5S2500 or D5S2800

Previously, two different loci, which are 1643 bp apart and have different sequence structure, were both named D5S2500. One locus is a part of the Investigator HDplex Kit (Qiagen) and the other one is a part of the AGCU 21-plex (AGCU ScienTech Incorporation). This duplication was detected when this locus showed different genotypes for the 9947A control DNA using both kits. Therefore, the name of D5S2800 was proposed for the STR marker included in the AGCU 21-plex to be differentiated from the one included in the Investigator HDplex Kit [19]. The Health Gene Technologies has confirmed that the D5 locus included in SureID 23comp Kit is the same locus in the AGCU 21-plex (personal communication). This was further confirmed by profiling the 9947A control DNA provided with the kit as a positive control. Therefore, the name of the locus has now changed nomenclature to D5S2800.

Table S 1. Bone samples used in the validation tests of the SureID 23comp Kit. Nine samples collected from a mass grave in Iraq were extracted using PrepFiler BTA Forensic DNA Extraction Kit (AB) and were quantified using Quantifiler Trio DNA Quantification Kit (AB). This table shows Quantifiler Trio small fragment concentrations (ng/µl), DIs, and total DNA quantities added to the PCRs of SureID Kit and of other kits. The percentages of detected alleles of autosomal STRs (aSTRs) when using different STR kits are also shown.

Sample #	**Quantifiler Trio**		**Total DNA added to PCRs:**	**% of detected alleles using different kits**			
	Small fragment concentration (ng/µl)	Degradation Index (DI)	SureID PCR (ng/6.25 µl)/Other kits (ng/15 µl)	SureID 23 (22 STRs)	PowerPlex 21 (20 STRs)	GlobalFiler (21 aSTRs)	PowerPlex Fusion 6C (23 aSTRs)
76 c	0.0173	57.666	0.1081/0.2595	27.30%	60%	66.60%	60.80%
78 a	0.0194	16.166	0.1213/0.2910	54.50%	90%	95.20%	82.60%
93 b	0.3271	2.7464	2.0444/4.9065	100%	100%	N/A	N/A
76 e	0.093	2.2962	0.5813/1.3950	100%	100%	N/A	N/A
81 a	0.0571	1.929	0.3569/0.8565	100%	100%	76.20%	N/A
97 b	0.0548	1.6758	0.3425/0.8220	100%	100%	N/A	N/A
94 a	0.0685	2.4204	0.4281/1.0275	100%	100%	N/A	N/A
25 a	0.0463	4.9784	0.2894/0.6945	86.30%	95%	N/A	N/A
46 b	0.0412	3.1937	0.2575/0.6180	100%	100%	N/A	N/A

N/A: sample was not profiled using this kit.

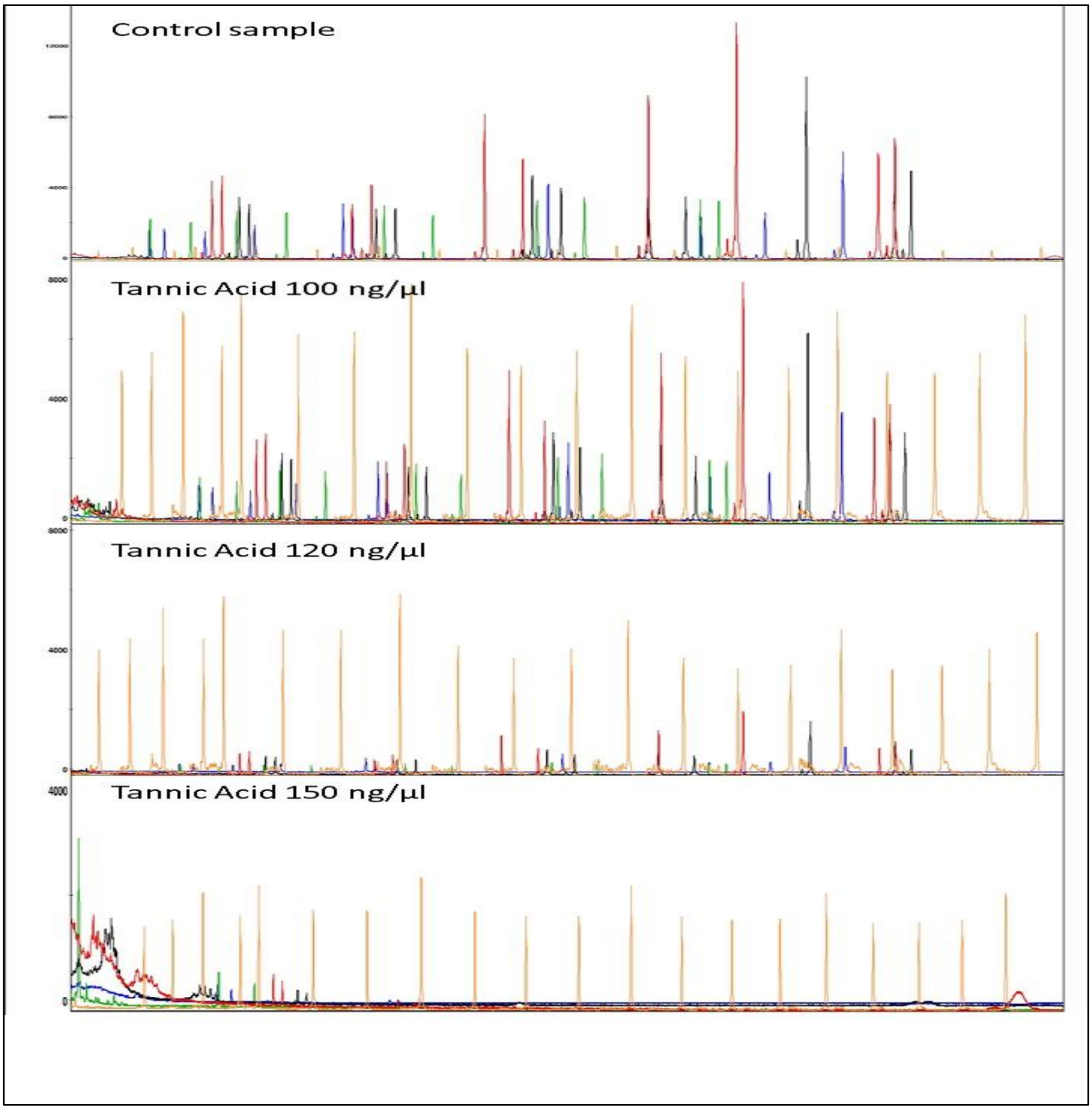

Figure S 1. Testing of the SureID 23comp Kit with tannic acid. Three different concentrations of (100, 120 and 150) ng/µl were tested. Full profiles were achieved with 120 ng/µl of tannic acid.

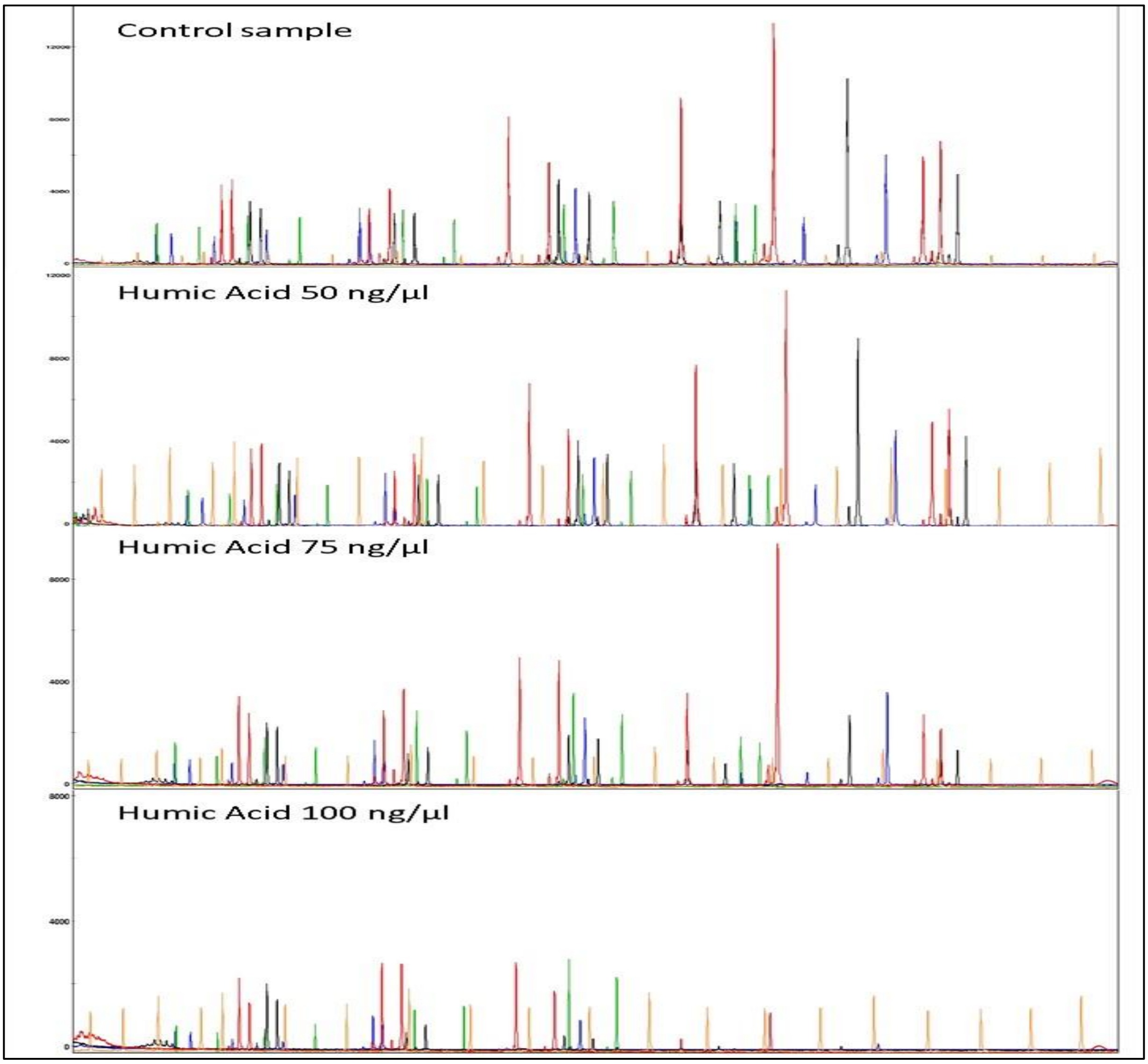

Figure S 2. Testing of SureID 23comp Kit with humic acid. Three different concentrations of (50, 75 and 100) ng/µl were tested. Full profiles were achieved with 75 ng/µl of humic acid.

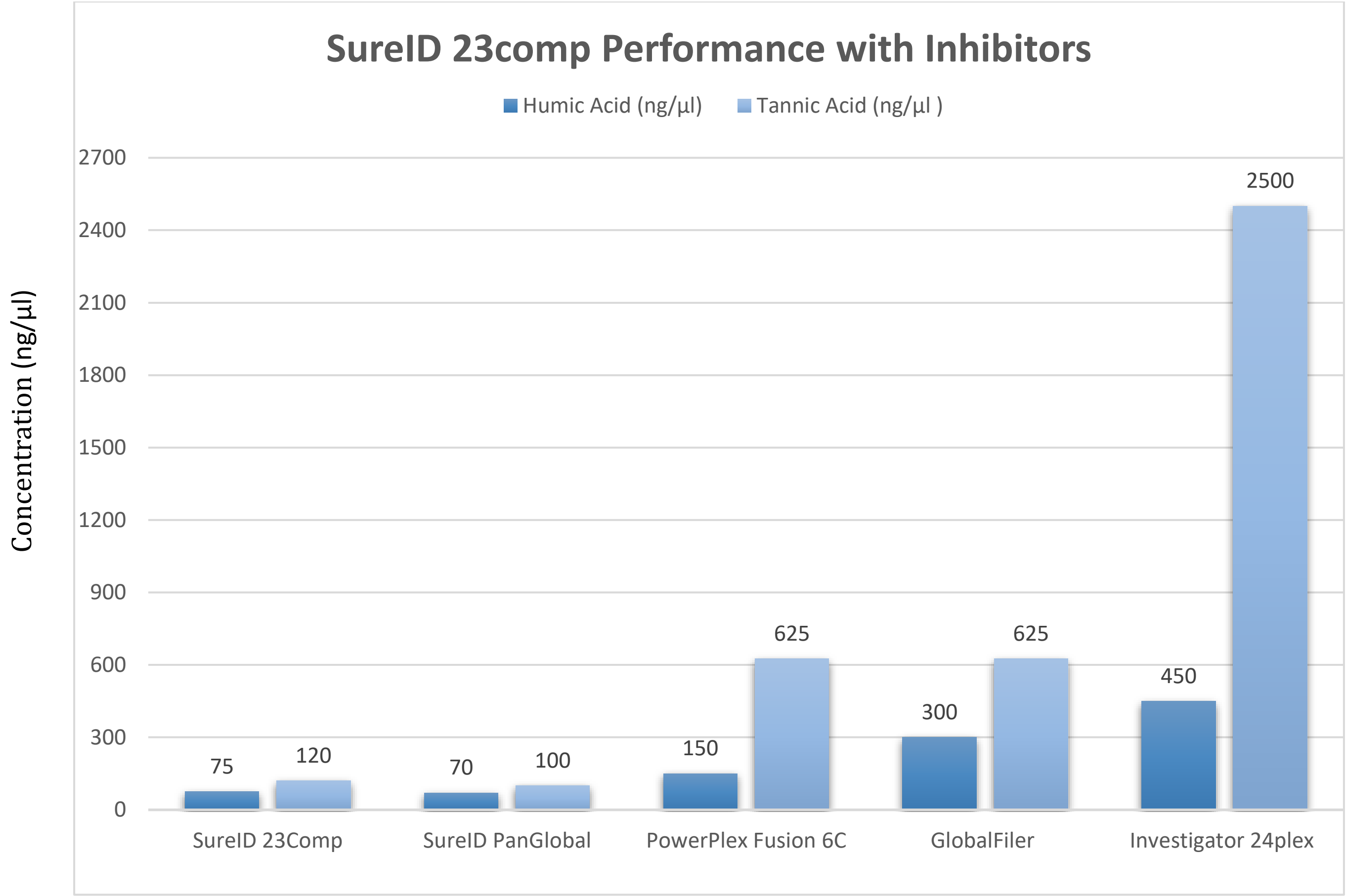

Figure S 3. SureID 23comp Kit performance with two common PCR inhibitors. Full profile was generated with the presence of 75 ng/µl of humic Acid and 120 ng/µl of tannic Acid. These figures are similar to those reported for the SureID PanGlobal Kit (Health Gene Technologies) [32]. However, the kit was not as robust with inhibitors as PowerPlex Fusion 6C, GlobalFiler, and Investigator 24plex [33].

Table S 2. PCRs contents for the SureID 23comp, PowerPlex 21. GlobalFiler, PowerPlex Fusion 6C. The table is showing the contents of the 25 µl volume PCRs for four kits used to genotype the bone samples. The SureID 23comp has less space (6.25 µl) for DNA input compared to the other three kits (15 µl). Increasing the concentration of the master and primer mixes will increase the space for the DNA input.

Kit	PCRs total volume	Master Mix	Primer Mix	Maximum DNA input
SureID 23comp	25 µl	12.5 µl	6.25 µl	6.25 µl
PowerPlex 21	25 µl	5 µl	5 µl	15 µl
GlobalFiler	25 µl	7.5 µl	2.5 µl	15 µl
PowerPlex Fusion 6C	25 µl	5 µl	5 µl	15 µl

Table S 3. Peak balance ratios study for the SureID 23comp Kit. A total of 90/500 samples were used to study balance ratios for the amelogenin (AMEL) and 22 STRs included in the SureID 23comp Kit. The 10 µl reaction volume was evaluated using three DNA quantities 0.5, 0.35, and 0.25 ng. The 0.5 ng showed the highest peak ratios average. The D21S2055 showed the lowest ratio at all DNA quantities (in bold).

Marker	Peak balance ratios (%) of the 10 µL reaction volume		
	0.5 ng	0.35 ng	0.25 ng
AMEL	94.29	83.90	81.83
D18S1364	90.90	86.11	83.90
D1S1656	86.62	87.43	85.21
D13S325	91.35	81.69	84.15
D5S2800	85.95	88.85	80.28
D9S1122	90.65	85.88	84.80
D4S2366	91.42	86.94	85.15
D3S1744	90.08	87.97	87.23
D12S391	85.38	83.09	78.35
D11S2368	91.57	84.19	83.81
**D21S2055**	**73.11**	**79.75**	**68.12**
D20S482	94.02	91.62	87.02
D8S1132	88.96	82.64	84.44
D7S3048	85.98	86.05	80.80
D2S441	90.83	84.08	87.61
D19S253	82.62	87.44	79.48
D10S1248	90.53	84.86	85.27
D17S1301	92.58	90.00	87.64
D22GATA198B05	86.48	85.54	80.41
D16S539	87.54	85.94	87.94
D6S474	85.07	87.73	84.22
D14S1434	88.41	87.93	85.99
D15S659	86.74	85.12	82.72
Average (%)	88.31	85.86	83.32

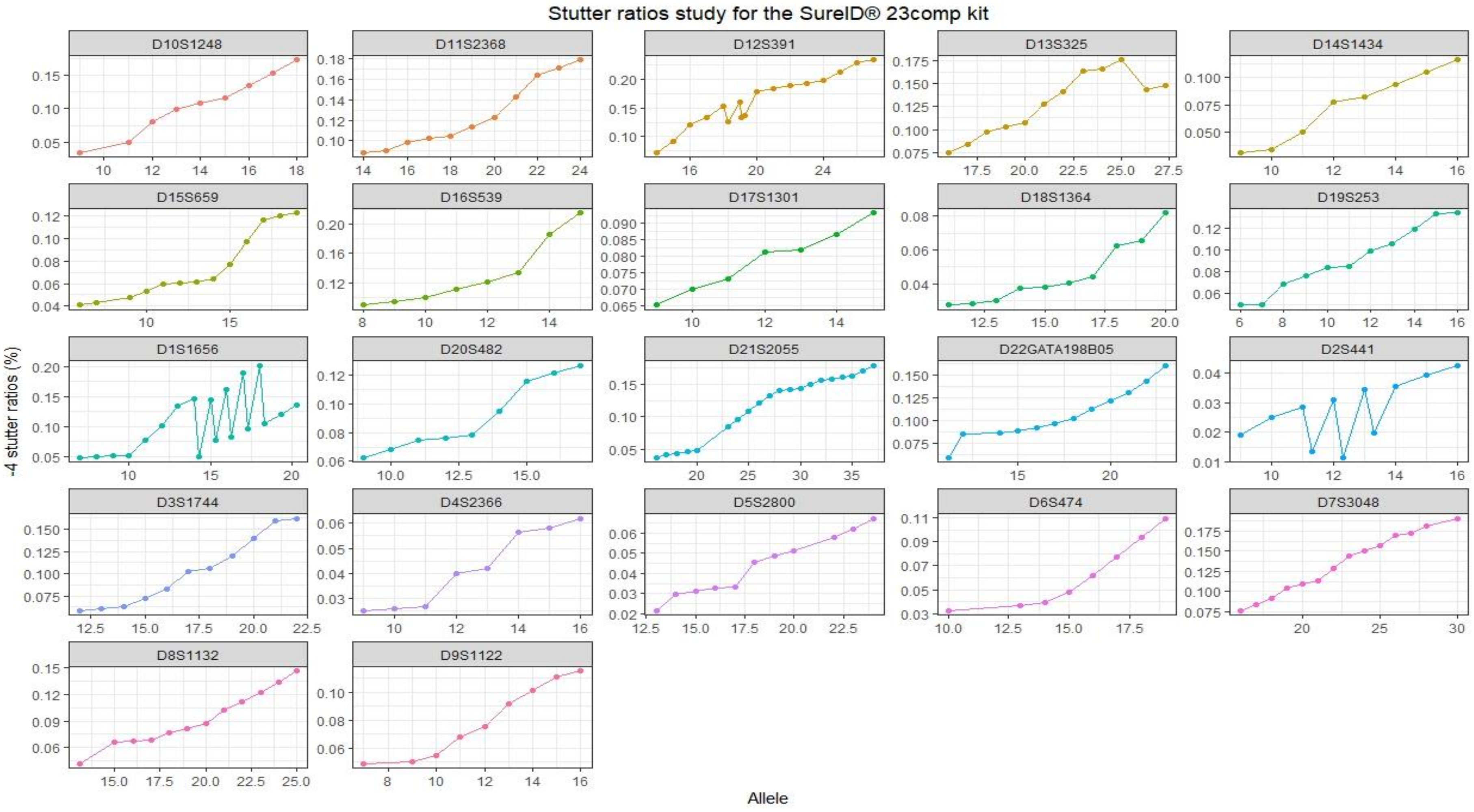

Figure S 4. Stutter ratios study for the SureID 23comp Kit. The figure shows the - 4 stutter ratios for STRs included in the SureID 23comp kit. Each box represents the stutter ratios of an STR. The line was drawn based on the average ratios of observed stutters. Alleles of x.1, x.2 and x.3 are plotted at x.25, x.5, and x.75 respectively. The average of stutter ratios ranged from 3.8% for D2S441 to 16.15% for D12S391.

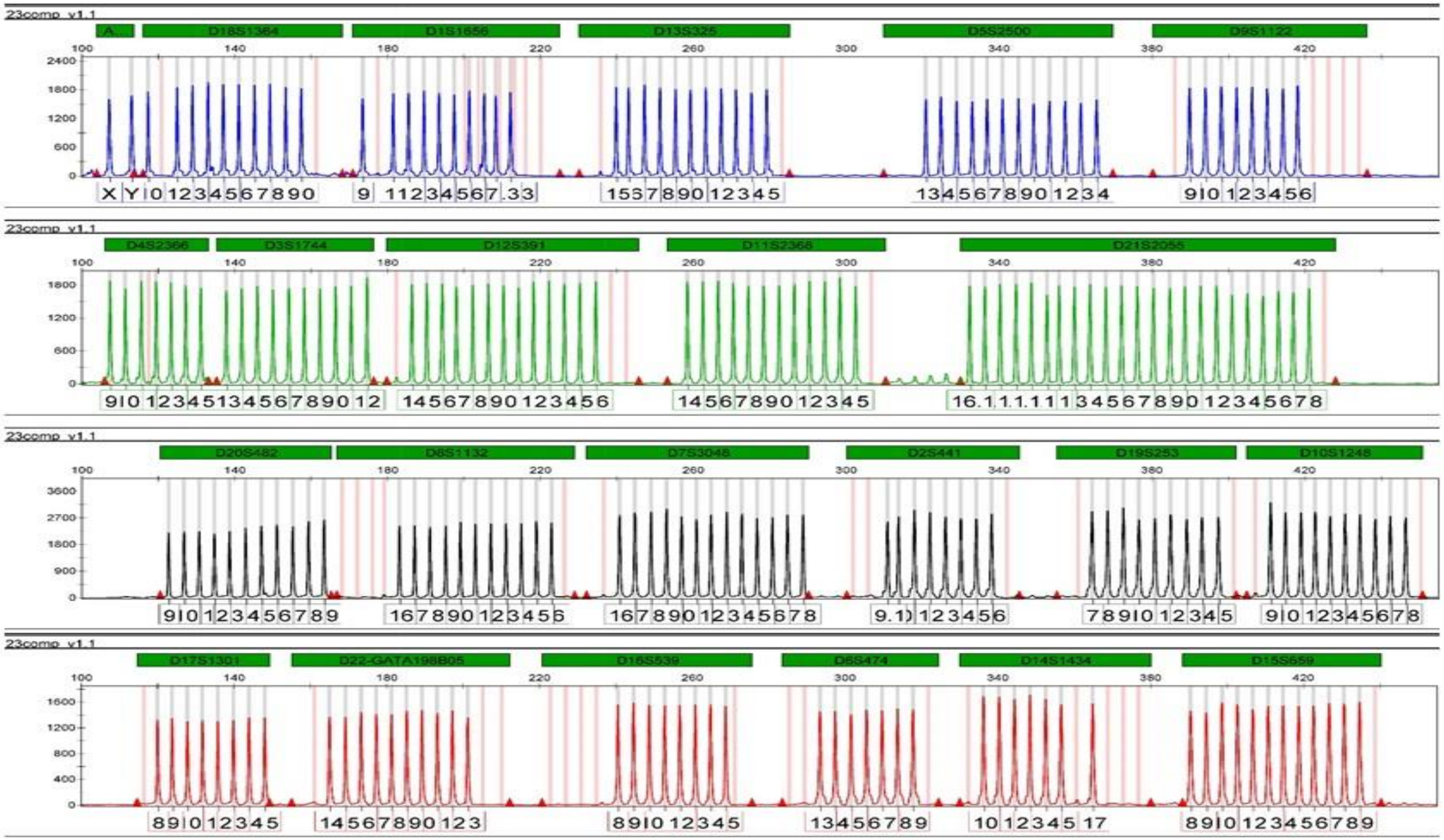

Figure S 5 Allelic ladder of the SureID 23comp Kit. This figure shows the allelic ladder provided with the SureID 23comp Kit. It represents 232 alleles that are supported by 53 additional bins for variant alleles (pink bins).

Table S 4. 38 STR loci included in the SureID 23comp & GlobalFiler kits. The loci are ordered based on their informativeness.

STRs order	GlobalFiler	SureID 23comp	Matching Probability
1	SE33		0.007
2		D21S2055	0.016
3	D12S391	D12S391	0.026
4		D7S3048	0.027
5	D1S1656	D1S1656	0.030
6	D19S433		0.030
7	D18S51		0.031
8	FGA		0.033
9	D2S1338		0.035
10		D8S1132	0.041
11		D22GATA198B05	0.045
12		D18S1364	0.046
13		D15S659	0.046
14	D8S1179		0.051
15	D21S11		0.055
16		D3S1744	0.060
17		D11S2368	0.068
18		D13S325	0.070
19		D4S2366	0.076
20	D7S820		0.076
21		D5S2800	0.079
22	vWA		0.082
23		D19S253	0.083
24	TH01		0.085
25	D16S539	D16S539	0.087
26	D13S317		0.087
27	D3S1358		0.091
28	D2S441	D2S441	0.091
29	D10S1248	D10S1248	0.098
30	D5S818		0.098
31		D6S474	0.102
32	CSF1PO		0.122
33	D22S1045		0.138
34		D9S1122	0.141
35		D20S482	0.143
36		D14S1434	0.148
37	TPOX		0.160
38		D17S1301	0.162
CMP	$1.421 \text{ X } 10^{-26}$	$7.4 \text{ X } 10^{-27}$	
CPE	0.999997405	0.999998692	
Theoretical MCP frequency	$2 \text{ X } 10^{-20}$	$3 \text{ X } 10^{-21}$	
CMP (38 loci)	$1.7 \text{ X } 10^{-46}$		
CPE (38 loci)	0.999999999934248		
Theoretical MCP frequency (38 loci)	$2.7 \text{ X } 10^{-36}$		

CMP: Combined Match Probability
CPE: Combined Power of Exclusion
MCP: Theoretical Most Common Profile assuming heterozygosity at all loci.

Table S 5. The maximum matching loci within the 500 samples. In the 500 samples, only two pairs of samples showed full matching in 9 loci (i.e. both alleles). This was the maximum number of matched loci. One pair of profiles showed partial matching (i.e. one of the two alleles) at 20 out of 22 loci. This table was generated by the R studio using the package of DNA tools.

No of Matched Loci	No of Partial Matched Loci																						
	**0**	**1**	**2**	**3**	**4**	**5**	**6**	**7**	**8**	**9**	**10**	**11**	**12**	**13**	**14**	**15**	**16**	**17**	**18**	**19**	**20**	**21**	**22**
**0**	0	0	2	11	29	114	320	730	1395	2219	3194	3689	3493	2820	2032	1065	494	194	57	19	1	0	0
**1**	0	0	0	19	90	309	768	1816	3296	5036	6509	6769	6069	4408	2663	1264	448	133	24	5	0	0	
**2**	0	1	2	26	114	389	1060	2212	3588	5336	6016	5716	4473	2852	1450	565	189	39	7	0	0		
**3**	0	0	4	22	98	333	792	1660	2456	3269	3452	2811	1927	1019	469	158	35	8	2	0			
**4**	0	0	3	18	68	179	409	757	1081	1299	1183	899	517	268	76	20	4	0	0				
**5**	0	0	0	12	20	83	179	272	348	364	278	194	124	26	7	0	1	0					
**6**	0	0	1	6	8	26	53	63	89	85	49	25	9	0	1	0	0						
**7**	0	0	1	0	1	8	11	17	24	15	6	1	1	1	0	0							
**8**	0	0	0	1	1	3	2	1	2	0	0	0	0	0	0								
**9**	0	0	0	0	0	0	0	0	1	0	0	1	0	0									
**10**	0	0	0	0	0	0	0	0	0	0	0	0	0										
**11**	0	0	0	0	0	0	0	0	0	0	0	0											
**12**	0	0	0	0	0	0	0	0	0	0	0												
**13**	0	0	0	0	0	0	0	0	0	0													
**14**	0	0	0	0	0	0	0	0	0														
**15**	0	0	0	0	0	0	0	0															
**16**	0	0	0	0	0	0	0																
**17**	0	0	0	0	0	0																	
**18**	0	0	0	0	0																		
**19**	0	0	0	0																			
**20**	0	0	0																				
**21**	0	0																					
**22**	0																						

Table S 6. An assessment of the SureID 23comp for kinship testing. This table shows the paternity probability for a typical paternity case by using combined typical paternity index for different prior probabilities (Pr = 0.90, 0.50 and 0.10). The SureID 23comp showed higher probabilities comparing to those probabilities calculated when using the GlobalFiler kit. It shows the paternity probability when using the 38 loci at different prior probabilities.

kit	Typical combined paternity index	Paternity probability (%)		
		Pr = 0.90	Pr = 0.50	Pr = 0.10
SureID 23comp	93835307.21	99.99999988	99.99999893	99.99999041
GlobalFiler	42569026.49	99.99999974	99.99999765	99.99997886
SureID 23comp & GlobalFiler kits	3.99448×10^{15}	99,9999999999999972	99,9999999999999750	99,9999999999997750

Table S 7. CMP of the SureID 23comp & GlobalFiler kits in different profiling scenarios. By combining the two kits, the total number of STR loci is 38 loci, 12 loci < 200 bp, and 8 loci < 150 bp.

	Complete profile		< 200 bp [b]		< 150 bp [c]	
	CMP	STRs #	CMP	STRs #	CMP	STRs #
SureID 23comp	7.154×10^{-27}	22	4.7×10^{-6}	5	7.8×10^{-5}	4
GlobalFiler	1.421×10^{-26}	21	1.7×10^{-8}	7	0.000111185	4
SureID 23comp & GlobalFiler	1.7×10^{-46}	38 [a]	7.9×10^{-14}	12	8.7×10^{-9}	8

[a]: Five common loci between the two kits (D1S1656, D12S391, D16S539, D2S441, and D10S1248).
[b]: SureID 23comp: D18S1364, D4S2366, D3S1744, D20S482, and D17S1301. GlobalFiler: D3S1358, D8S1179, D2S441, D19S433, D22S1045, D5S818 and D10S1248.
[c]: SureID 23comp: D18S1364, D4S2366, D20S482 and D17S1301. GlobalFiler: D3S1358, D2S441, D22S1045 and D10S1248.
